# Encephalitis and myositis caused by *Trachipleistophora hominis* diagnosed by metagenomic next-generation sequencing—a case report

**DOI:** 10.3389/fcimb.2023.1206624

**Published:** 2023-07-31

**Authors:** Haipeng Zheng, Ying Tan, Xudan Chen, Jinfeng Chen, Linghua Li, Jian Wang

**Affiliations:** Infectious Disease Center, Guangzhou Eighth People’s Hospital, Guangzhou Medical University, Guangzhou, Guangdong, China

**Keywords:** microsporidia, *Trachipleistophora hominis*, MNGs, myositis, brain infection

## Abstract

**Background:**

Myositis is the main manifestation of *Trachipleistophora hominis* (*T. hominis*) infection and other microsporidians infection in immunocompromised patients. Clinical differential diagnosis of different microsporidians can be challenging, as the standard technique to distinguish various microsporidia species, transmission electron microscopy (TEM), is time-consuming and relies on equipment and experienced staffs who can perform the test and interpret the results.

**Case presentation:**

We report a 37-year-old Chinese man with acquired immune deficiency syndrome (AIDS) developed headache and muscle pain in the extremities. Tramadol was used to relieve his pain. Infectious lesions in his brain were detected by cerebral magnetic resonance imaging (MRI). Oval-shaped pathogens was observed by biopsy of right gastrocnemius. Finally, *T. hominis* was identified by metagenomic next-generation sequencing (mNGS) in the gastrocnemius tissue and cerebrospinal fluid. After a 12-week course of antifungal treatment and antiretroviral therapy, the patient recovered from the encephalitis and myositis caused by *T. hominis.*

**Conclusion:**

This report described the diagnosis and treatment of the first case of encephalitis caused by *T. hominis.* And mNGS is recommended for the rapid diagnosis of uncommon pathogens.

## 1 Introduction

Microsporidia, the causative agent of microsporidiosis, is a group of intracellular parasites which are capable of infecting various of invertebrate and vertebrate hosts. Microsporidia encompasses 187 named genera with approximately 1500 described species, of which only 17 species are reported to be pathogenic to humans (Han et al., 2021).

Microsporidia are widespread in nature, but before the recognition of human immunodeficiency virus (HIV) infection, such opportunistic infections were rarely found in humans. Microsporidial myositis is first reported in association with infection HIV in 1985 (Ledford et al., 1985). With the increasing number of HIV patients, the harm of microsporidia to humans has attracted more attention. Currently, few case reports of *T. hominis* infection in humans have been reported worldwide. In the case, we reported a microsporidiosis infection in an acquired immune deficiency syndrome (AIDS) patients in China which is the first brain infection of *T. hominis*.

## 2 Methods

### 2.1 Gomori methenamine silver staining assay

The slide was oxidized with 4% aqueous chromic acid at room temperature for 20 min and washed by water for a few seconds. Then, the slide was stained with 0.5% sodium metabisulphite for 1 min and washed by running water for 5 min and rinsed thoroughly in distilled water. The slide was then placed in silver solution in 60°C incubator for 15~30 min. When turned dark brown, the slide was rinsed with distilled water. A 0.1% gold chloride solution was added to the slide and incubated for 2 min followed by rinsing well with distilled water. The slide was then treated with 2% sodium thiosulphate for 3 min and then washed under smoothly running tap water for 5 min. Eosin solution was then used to counterstain the slide for 1~2 s and then rinsed to remove the excess alcohol.

### 2.2 Fungal fluorescence staining assay

Fungal fluorescence staining was performed according to the instruction of a Fungal Fluorescence Staining Kit (GongyingLian Biotechnology, Jiangsu, China). Briefly, the slide was stained with fungal fluorescence solution for 1 min, and then observed by fluorescence microscope.

### 2.3 Next-generation sequencing and data analysis

DNA libraries for next-generation sequencing were constructed from extracted nucleic acid derived from gastrocnemius or cerebrospinal fluid samples, followed by library validation and sequencing on an Illumina MiSeq instrument. Reads were subtracted human host sequences, followed by alignment of reads to reference sequences in databases for the identification of pathogens.

## 3 Case presentation

On 24 January 2022, a 37-year-old male office clerk presented to our hospital for investigation and management of muscle pain in the extremities with headache. The man used to be in good health and did not have the habits of smoking and alcohol. Five months prior to his admission, he began suffering from symmetry pain in the muscles of the limbs, accompanied by headache on the left side, without nausea, vomiting, or fever. Physical examination revealed body temperature of 36.4°C, heart rate of 76 beats per minute, and blood pressure of 117/69 mm Hg; marked tenderness in gastrocnemius, quadriceps and biceps brachialis. Cardiovascular, abdominal, and neurological examination was unremarkable except for weakness and stiffness in the extremities.

Blood chemistry tests revealed myositis marked by lactate dehydrogenase (LDH) of 695 U/L (normal range, 120-450 U/L), creatine kinase (CK) of 1955 U/L (normal range, 50-310 U/L), creatine kinase-MB (CK-MB) of 438 U/L (normal range, 0-24 U/L), myoglobin (MYO) of 900 U/L (normal range, 23-112 U/L). Preliminary etiological investigations excluded the presence of necrotizing autoimmune myopathy, cryptococcosis, tuberculosis, and toxoplasmosis, but Human immunodeficiency virus (HIV) RNA was positive (2.55×10^6^ copies/μL). Subsequent flow cytometry showed the CD4+ T cell count is 6 cells/µL.

**Figure 1:**
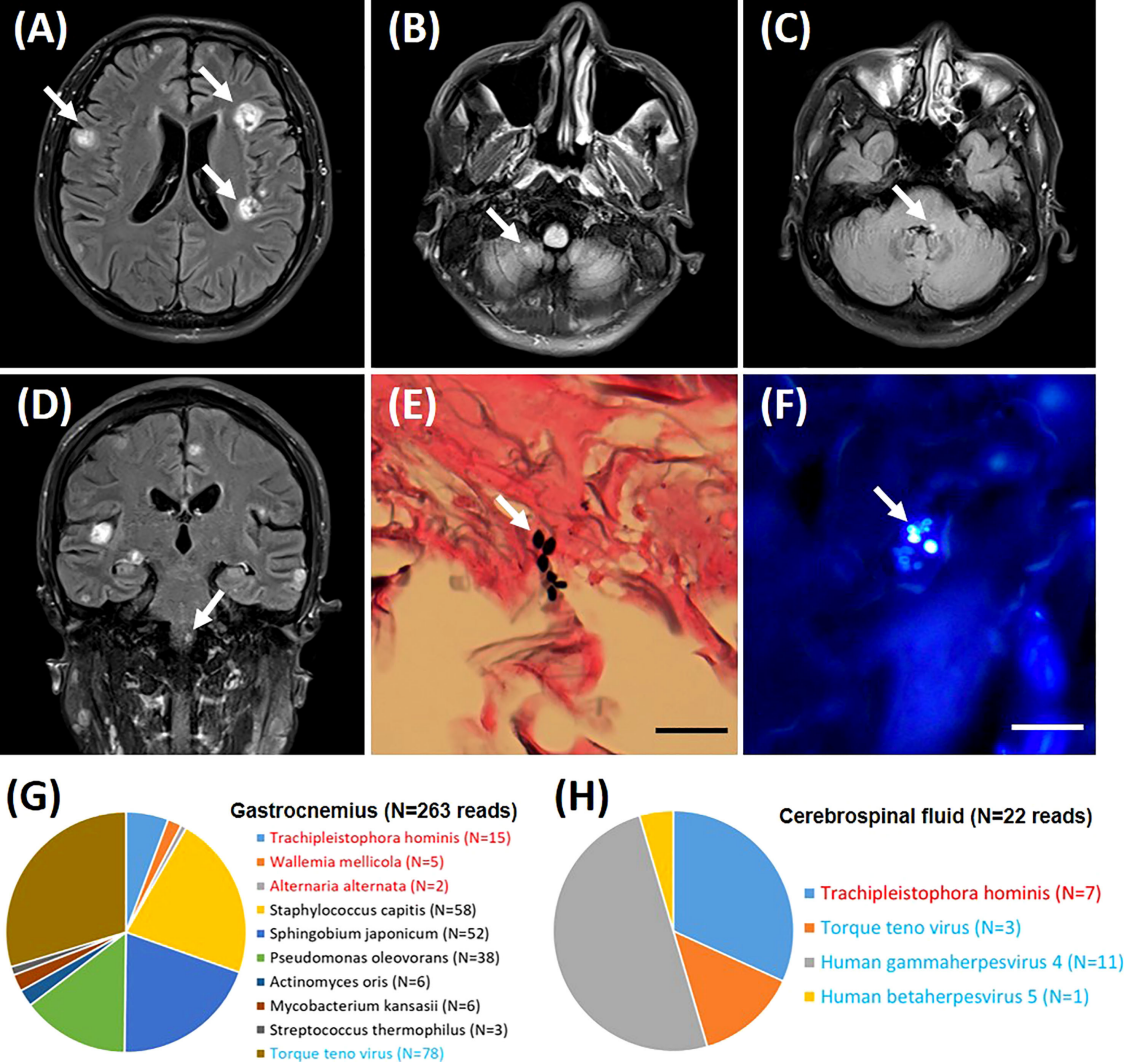
Imaging and pathological diagnosis. Brain magnetic resonance imaging (high signal on the T_2_ weighted images) shows multiple nodules in bilateral cerebral hemispheres **(A)**, right cerebellar hemispheres **(B)**, pons **(C)**, and medulla oblongata **(D)**. Histopathology demonstrates spores of *Trachipleistophora hominis* in sections of gastrocnemius muscle stained with Gomori methenamine silver **(E)** and fungal fluorescence staining **(F)**. Lesions and pathogens were marked by arrows. Pathological images are shown at ×400 magnification. Bar=20 μm. The distribution of fungal, bacterial and viral sequences identified in the patient’s Gastrocnemius **(G)** and Cerebrospinal fluid **(H)**. The name of the fungi and viruses are marked in red and blue, respectively.

Cerebral magnetic resonance imaging (MRI) revealed multiple parenchymal lesions with fine contrast enhancement in bilateral cerebral hemispheres, pons, medulla oblongata, and right cerebellar hemispheres, most of which are located in bilateral cerebral cortex (the large one can reach 15 mm in diameter) (**Figures 1A–D**). The MRI results suggested infectious lesions. A biopsy of right gastrocnemius was performed, and pathology showed infiltration of lymphocytes and neutrophils. Several scattered oval-shaped pathogens were observed by Gomori methenamine silver staining (**Figure 1E**) and fungal fluorescence staining (**Figure 1F**). To determine the a etiological agents, the gastrocnemius tissue and cerebrospinal fluid were subjected to metagenomic next generation sequencing (mNGS), respectively. Within 24 hours after receipt of the samples, 3 species of fungi, 6 species of bacteria, and 1 species of virus were detected in the gastrocnemius tissue (**Figure 1G**). Meanwhile, 1 species of fungi and 3 species of viruses were detected in cerebrospinal fluid sample (**Figure 1H**). Among these microbes, only *T. hominis* has been reported to cause myositis in immunocompromised patients. And *T. hominis* was identified in both gastrocnemius tissue and cerebrospinal fluid. Finally, the diagnosis of myositis and brain infection caused by the infection of *T. hominis* was made.

**Figure 2:**
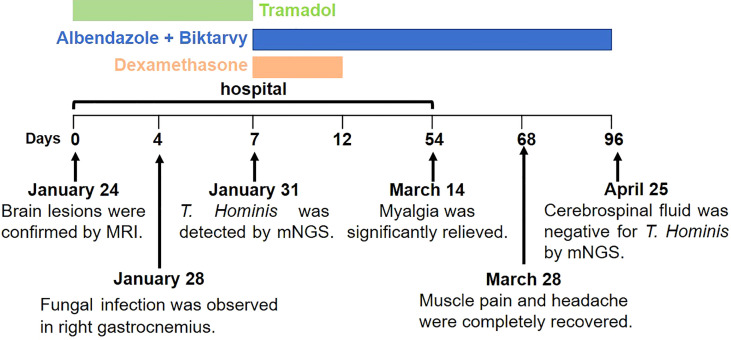
The timeline of disease progression and treatment of the patients.

During the hospitalization, he received tramadol to relieve his pain. After the *T. hominis* infection was confirmed, he initiated a 12-week course of oral albendazole (800 mg/d), a 5-day course of oral dexamethasone (5 mg/d), and regular antiretroviral therapy (Biktarvy). The patient was discharged after 6 weeks of treatment, when the myalgia was significantly relived, and the size and degree of brain lesions were improved (**Figure S1**). Upon follow-up in 2 weeks, the muscle pain and headache were completely recovered. After the 12-week course of treatment, mNGS showed that his cerebrospinal fluid was negative for *T. Hominis* (**Figure 2**).

## 4 Discussion

Microsporidia can cause localized or disseminated infection in humans. Currently, approximately 20 cases of disseminated microsporidiosis have been reported worldwide. The vast majority of these cases are distributed in America, Europe, and Oceania until 2021 when two cases of microsporidial myositis were reported in Thailand, Southeast Asia (Buppajarntham et al., 2021; Siripaitoon et al., 2021; Seatamanoch et al., 2022). Most recently, a case of microsporidial myositis caused by *Anncaliia algerae* Microsporidiosis in a kidney transplant recipient was reported in China (Liu et al., 2022). *T. hominis* was initially identified in an AIDS patient with myosotis in 1996 and has caused eight cases of human infection (Field et al., 1996; Seatamanoch et al., 2022). Myositis is the most common clinical manifestation for *T. hominis*, which is corresponded to the fact that muscle tissue is the most frequently (7 out of 8 cases) involved in these cases (Seatamanoch et al., 2022). Our patient suffered from severe myositis in the extremities, and skeletal muscle (gastrocnemius) infection was demonstrated by histopathology and mNGS. Although not confirmed by biopsy, myocardial damage was also suspected because of elevated CK-MB. This speculation can be supported by an autopsy report where *T. hominis* was found in the myocardium and pectoral muscles of a patient with advanced AIDS (Curry et al., 2005). In addition to muscle tissue, many other organs were reported to be involved in the infection of *T. hominis*, which include sinuses, testis (Ledford et al., 1985), eye stroma (Rauz et al., 2004), and bone marrow (Buppajarntham et al., 2021). Of note, in this case, brain infection was proved by cerebral MRI and mNGS of cerebrospinal fluid. Our report first confirmed that *T. hominis* can establish infection in central nervous system and caused brain damage.

Light microscopic (LM), transmission electron microscopy (TEM), and polymerase chain reaction (PCR) was commonly used for the diagnosis of microsporidiosis. Most recently, mNGS has also been utilized for the diagnosis of microsporidiosis in a transplant recipient (Liu et al., 2022). TEM is the standard technique for distinguishing different microsporidia species by observing the ultrastructural characteristics. However, this technique is not widely utilized due to the high requirements on equipment and experienced staffs who can perform the test and interpret the results (Ramanan and Pritt, 2014). Untargeted mNGS has the advantage of detecting all potential pathogens within a shorter time (24~48 h), which is more suitable for the identification of unexpected or unknown organisms (Chiu and Miller, 2019). In our case, fungi were observed in gastrocnemius tissue by LM, but the genus and species cannot be determined. Then, the pathogen, *T. homini*, was further confirmed by mNGS within one day.

Immune status is closely related to the consequence of microsporidial infection. In contrast to immunocompetent individuals, immunocompromised patients (such as AIDS patients, transplant recipients, and cancer patients) are more susceptible to microsporidial infection. Out of eight *T. hominis* cases, seven cases were found in AIDS patients (Seatamanoch et al., 2022). On admission, the cellular immunity of our patient is severely compromised (CD4+ T cell level is 6 cells/µL), which is an important reason for the extensive and persistent infection in muscles and brain. Therefore, effective antiretroviral therapy and immunological reconstitution were of significant importance for treatment of microsporidiosis in AIDS patient.

In conclusion, we reported the first case of microsporidiosis in an AIDS patient in China and also described the first brain infection of *T. hominis*. And mNGS is recommended for the rapid diagnosis of uncommon pathogens.

## Data availability statement

The original contributions presented in the study are included in the article/supplementary material. The sequencing data presented in the study are deposited in the Bioproject database (https://ngdc.cncb.ac.cn/bioproject/browse/PRJCA018308). The accession number is PRJCA018308. Further inquiries can be directed to the corresponding authors.

## Ethics statement

Written informed consent was obtained from the individual(s) for the publication of any potentially identifiable images or data included in this article. Written informed consent was obtained from the participant/patient(s) for the publication of this case report.

## Author contributions

JW and LL contributed to the study design and the manuscript preparation, and HZ and YT contributed to data analysis. XC and JC contributed to the collection of clinical specimens and information. All authors read and approved the final manuscript.

## Supplementary material

Supplementary Figure 1 Comparison of brain magnetic resonance imaging images before (**A–C**) and after treatment (**D–F**).
