# Supplementary figures and images for: Encephalitis and myositis caused by Trachipleistophora hominis diagnosed by metagenomic next-generation sequencing—a case report
Source: Front Cell Infect Microbiol. 2023 Jul 31;13:1206624. doi: 10.3389/fcimb.2023.1206624 (PMC10423897; doi:10.3389/fcimb.2023.1206624)

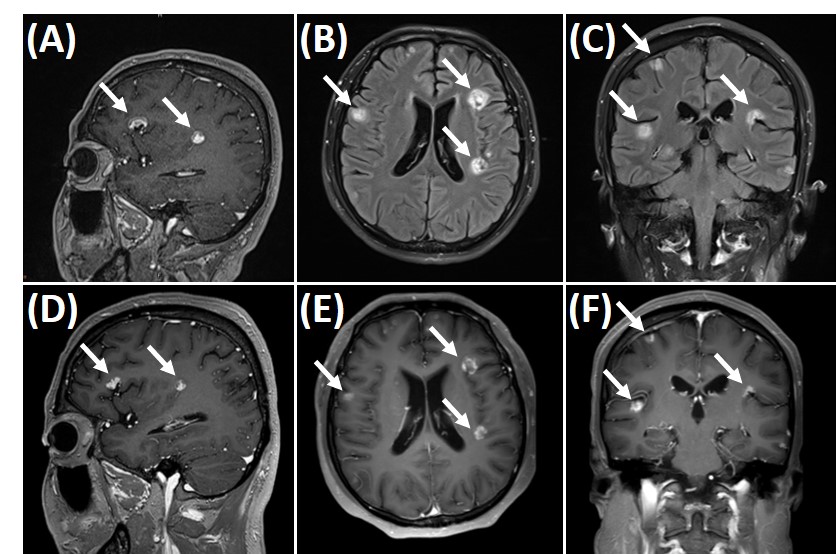

Supplement: Supplementary Figure 1 — Comparison of brain magnetic resonance imaging images before (A–C) and after treatment (D–F). [file Image_1.jpeg]
